# Comprehensive mapping of antigen specific T cell responses in hepatitis C virus infected patients with or without spontaneous viral clearance

**DOI:** 10.1371/journal.pone.0171217

**Published:** 2017-02-07

**Authors:** Chao Zhang, Rui Hua, Yuanyuan Cui, Shasha Wang, Hongqing Yan, Dongmei Li, Yonghong Zhang, Zhengkun Tu, Pei Hao, Xinyue Chen, Jin Zhong, Junqi Niu, Xia Jin

**Affiliations:** 1 Viral Disease and Vaccine Translational Unit, Institut Pasteur of Shanghai, Chinese Academy of Sciences, Shanghai, China; 2 Department of Hepatology, The First Hospital, Jilin University, Changchun, Jilin, China; 3 Department of Hepatology, Beijing You'an Hospital, Capital Medical University, Beijing, China; 4 Institute of Translational Medicine, The First Hospital, Jilin University, Changchun, Jilin, China; 5 Bioinformatics Core, Institut Pasteur of Shanghai, Chinese Academy of Sciences, Shanghai, China; 6 Viral Hepatitis Unit, Institut Pasteur of Shanghai, Chinese Academy of Sciences, Shanghai, China; Saint Louis University, UNITED STATES

## Abstract

Elucidating protective immunity against HCV is important for the development of a preventative vaccine. We hypothesize that spontaneous resolution of acute HCV infection offers clue to protective immune responses, and that DAA therapy affects the quality and quantity of HCV-specific T cell responses. To test these hypotheses, we performed T cell epitope mapping in 111 HCV-infected individuals including 61 chronically HCV-1b (CHC-1b) infected, 24 chronically HCV-2a (CHC-2a) infected and 26 spontaneously recovered (SPR) patients with 376 overlapping peptides covering the entire HCV polyprotein. Selected T cell epitopes were then used to evaluate T cell responses in another 22 chronically HCV-1b infected patients on DAA therapy. Results showed that SPR had better HCV-specific T cell responses than CHC, as manifested by higher response rate, greater magnitude and broader epitope coverage. In addition, SPR recognized novel epitopes in Core, E1, E2, NS4B, NS5A regions that were not present in the CHC. Furthermore, during the first 24 weeks of DAA therapy, there was no functional immune reconstitution of HCV-specific T cells. These results indicate that T cell responses may be a correlate of protection. Therefore, effective preventative vaccines should elicit a robust T cell response. Although various DAA regimens efficiently cleared viruses from the blood of HCV-infected patients, there was no contemporaneous early T cell immune reconstitution, suggesting that early treatment is needed for preserving the functions of HCV-specific T cells.

## Introduction

Over 185 million people globally are chronically infected by hepatitis C virus (HCV), approximately 0.5 million deaths occur each year as a result. HCV can be classified into 7 genotypes. Among them, genotype 1 is the most common and accounts for nearly 50% of global infections. HCV belongs to the *Flaviviridae* family of a single positive stranded RNA virus. Its genome encodes three structural proteins (core, E2, E2) and seven non-structural proteins (p7 (NS1), NS2, NS3, NS2A, NS4B, NS5A, NS5B). Acute HCV infection leads to either spontaneous viral clearance or persistent infection, which may progress to liver cirrhosis and hepatocellular carcinoma [1].

Host immune responses during the acute phase of HCV infection may determine the disease outcome. A rapid induction of neutralizing antibody (nAb) response in the acute phase was associated with virological control in some studies [2], but the elimination of viral RNA was also detected in patients without high-titer nAb responses [3]. Additionally, viremia persists in chronic HCV infection even in the presence of high levels of nAb, due possibly to the high frequency of viral escape mutations from neutralization [4]. These observations led to the postulate that immune factors other than antibodies may play a protective role. T cells have been demonstrated to be important in the control of many viral infections. Vigorous and poly-functional HCV-specific T cell responses have been associated with the spontaneous clearance of infection, whereas the absent or weak T cell responses have often been observed in chronic infection [5]. In acute HCV infection, functional, strong and broad CD4+ responses have been linked to the resolution of infections, better maturation of memory CD8+ T cells [6], and rapid viral load reduction [7]. In chronic HCV infection, CD4+ T cells were rapidly exhausted, with a marked loss of interleukin-2 (IL-2) production and proliferative capacity, but an increased IL-10 production [8]. There are also exhausted CD8+ T cells that lacked antigen-specific T-bet induction, but had an increased surface expression of programmed death 1 (PD-1) molecules [9]. Also, mutational escape within viral specific CD8+ T cell epitopes has been found in patients who manifested a failure of virological control [10].

Recently, direct-acting antiviral (DAA) drugs, used either alone or in combination with interferon α (IFN-α), have achieved sustained virological response (SVR) in over 90% of treated patients [11]. Several DAA based therapies have been initiated to facilitate the introduction of these highly effective DAA into China. However, traditional therapy with PEGylated IFN-α (PEG-IFN-α) and ribavirin (RBV) that accomplished a 50% SVR rate is still the first line drugs for Chinese patients. How might DAA and other forms of antiviral regiments affect T cell immunity is still controversial. In some reports, the impaired T cell immune responses in chronic HCV-infected patients were partially reconstituted by DAA therapy with a restoration of HCV-specific CD8+ T cell function and antigen-stimulated memory T-cell differentiation [12]. In contrast, a restoration of specific T cell functions was not detected in patients who achieved SVR with PEG-IFN-α and RBV therapy [13]. Also, therapeutic vaccines failed to restore T cell immune responses in SVR patients, which implied that T cell exhaustion might have occurred in some patients during persistent HCV infection [14, 15].

In China, HCV subtype 1b (HCV-1b) is the most prevalent genotype which accounts for over 50% of infections, followed by 24% of infection caused by subtype HCV-2 [16]. There are several hot spots in mainland China where a large number of cases of HCV infections were detected, including Jilin province, Hebei province, Henan province, Guangdong province, and megacities such as Beijing and Tianjin. However, no study has systematically compared T cell responses across the entire length of HCV polyprotein between patients who experienced spontaneously resolution of infection and those who became chronically infected. Additionally, no comprehensive study on T cell function has been performed in Chinese patients on DAA therapy.

We hypothesized that spontaneous resolution of acute HCV infection offers clue to protective immunity and that DAA therapy affects the quality and quantity of HCV-specific T cell responses. To test these hypotheses, we systematically investigated T cell responses in spontaneously recovered (SPR) patients, chronically hepatitis C (CHC) infected patients and DAA treated patients using a full-genome synthetic peptide library of HCV-1b. Our results demonstrated striking differences in HCV-specific T cell responses between SPR and CHC, and revealed unique epitopes recognized by SPR but not CHC. Furthermore, although various DAA regimens efficiently cleared viruses from the blood of chronically HCV-infected patients, they did not lead to a contemporaneous early T cell immune reconstitution, suggesting that early treatment is needed for preserving T cell functions.

## Materials and methods

### Study subjects

A total of 111 HCV-infected DAA treatment-naive patients, including 61 chronic HCV-1b infected (CHC-1b), 24 chronic HCV-2a infected (CHC-2a) and 26 spontaneously recovered (SPR, defined as HCV specific antibodies positive, HCV RNA negative and treatment naive) individuals were recruited for the T cell epitope mapping study. These individuals were either recruited from a cohort established previously in Fuyu city, Jilin province, China [17], or recruited from two hospital clinics; one at the First Hospital of Jilin University in Jilin province, and the other at Beijing You'an Hospital of Capital Medical University in Beijing, China. PBMCs of 3 healthy control (HC) were purchased from a local hospital blood bank.

Another 22 DAA-treated CHC-1b patients were recruited from three clinical trials completed at the First Hospital of Jilin University. AI447-114 (ChinaDrugTrials.org.cn, identifier CTR20150462) is a 24-week interferon-free DAA therapy clinical trial with daclatasvir/asunaprevir; M13-767 (identifier CTR20150588) is a 12-week interferon-free DAA therapy clinical trial with paritaprevir/ombitasvir/dasabuvir/ritonavir; ASC08 (identifier CTR20150846) is a 12-week clinical trial with danoprevir/ritonavir/PEG-IFN-α2a/RBV. All subjects were tested negative for human immunodeficiency virus and hepatitis B virus, and had no clinical evidence of liver cirrhosis.

### Ethics statement

All human subject studies were approved by the ethics committee of Institut Pasteur of Shanghai (identifier IPS-2013017). Clinical studies were also approved by local ethics committee of the First Hospital of Jilin University (identifier 2014–193) and Beijing You'an Hospital of Capital Medical University (identifier 2014–24). All participants signed an informed consent.

### HCV peptides

Three hundred nighty-two complete HCV-1b genome sequences available in the NCBI nucleotide database and HCV database (hcv.lanl.gov, 2012) were downloaded. Open reading frame of each of these 392 sequence was translated, aligned to generate a 3,010 amino acids long consensus sequence which was used as the template for synthesis of 376 overlapping peptides (each is 18 amino acids in length, overlapping by 10 amino acids) covering the entire length of HCV polyprotein (purity 90%, GL Biochem, Shanghai). Peptides were dissolved in dimethyl sulfoxide (DMSO, SIGMA), diluted in PBS, mixed into 39 peptide pools (20 peptides in each pool), and then used as a 20×19 peptide matrix in the enzyme-linked immunospot (ELISPOT) assay as described previously [18].

### IFN-γ ELISPOT assay

*Ex vivo* IFN-γ ELISPOT assay was performed according to the manufacturers’ instruction (Mabtech, Sweden) with freshly isolated peripheral blood mononuclear cells (PBMCs). Briefly, PBMCs were isolated by Ficoll gradient centrifugation using venous blood, reconstituted in RPMI 1640 supplemented with 100U/ml penicillin, 100μg/ml streptomycin and 10% FBS (GIBCO), and then aliquoted at 2×10^5^cells/well in a 96-well PVDF plate (Merck Millipore). Followed by stimulation for 48hrs with either peptide pools (2μg/ml of each peptide), or phytohemagglutinin (PHA, SIGMA, 20μg/ml) as positive control, and DMSO alone (equivalent concentration to peptide pools) as negative control. Anti-CD49d and anti-CD28 (eBiosciences, 0.5μg/ml) antibodies were added to each well to provide co-stimulation. IFN-γ expression was detected with a biotinylated detection mAb (7-B6-1), streptavidin-HRP and TMB substrate (Mabtech, Sweden). Spot-forming units (SFUs) were counted with an ELISPOT plate reader (CTL Europe Gmbh). The definition of a HCV-specific T cell response was made based on results of healthy controls subjects who had no clinical history of HCV infection (S2B Fig). The mean (+3SD) value of spot forming units (SFUs) in all peptide stimulated wells of healthy controls was 64 SFUs/million. For simplicity in calculation, the following criteria were used as a cut-off for positivity: (i) the absolute SFU must be greater or equal to mean plus 3SD of the healthy controls; and (ii) the number of spots must be greater or equal twice the average value of the blank controls [19–21]. Each positive peptide response was given an arbitrary score of 100 for easy comparison among patient groups.

### Intracellular Cytokine Staining (ICS)

Immunodominant HCV-1b epitopes identified from SPR in the epitope mapping assay, including 21 peptides within the structural proteins and 18 peptides within the non-structural proteins were arranged into two peptide pools, structural protein peptide pool (SPP) and non-structural protein peptide pool (NSPP). ICS assay for IL-2 and IFN-γ were performed as described previously [22]. Freshly isolated PBMCs derived from 5 patients in the ASC08 clinical trial were stimulated with either SPP, NSPP (2μg/ml of each peptide), or PMA (SIGMA, 50ng/ml) combined with ionomycin (SIGMA, 1μg/ml) as positive control and DMSO alone as negative control for 6 hours at 37°C. GolgiPlug protein transport inhibitor (BD Biosciences, 1μg/ml), anti-CD49d and anti-CD28 (eBiosciences, 1μg/ml) antibodies were added along with the stimulation. Anti-PD-1 antibody (gift from Andy Tsun, Innovent Biologics, Inc. Suzhou, China) was added in some assays as needed. Cells were harvested, permeabilized and fixed using permeabilzation buffer (BD Biosciences), and then stained with CD3 (clone UCHT1), CD4 (clone SK3), CD8 (clone SK1), IL-2 (clone MQ1-17H12), IFN-γ (clone B27), PD-1 (clone NAT105), Ki67 (clone Ki67), CD38 (clone HB-7) and IL-10 (clone JES3-9D7) (BioLegend). Cell viability was evaluated with Live/Dead-Aqua (Life technologies). Stained cells were analyzed using LSR-Fortessa flowcytometer (BD Biosciences) and the FlowJov10.0.7 software, a minimal of 100, 000 lymphocytes were collected for each sample.

### Statistical analysis

In the HCV epitope mapping study with the IFN-γ ELISPOT assay, each peptide pool that stimulated a positive response was recorded, and a single positive peptide within a peptide pool was determined based on the peptide matrix method as described [18]. Each selected positive peptide was given a score of 100 in every ELISPOT assay to standardize the analyses. All statistical analysis was performed with the GraphPad Prism 6.01 software. Statistical differences between groups were determined by Student’s t test. A p value of 0.05 was considered statistically significant.

## Results

### Stronger T cell responses correlated with better clinical outcome

**Fig 1:**
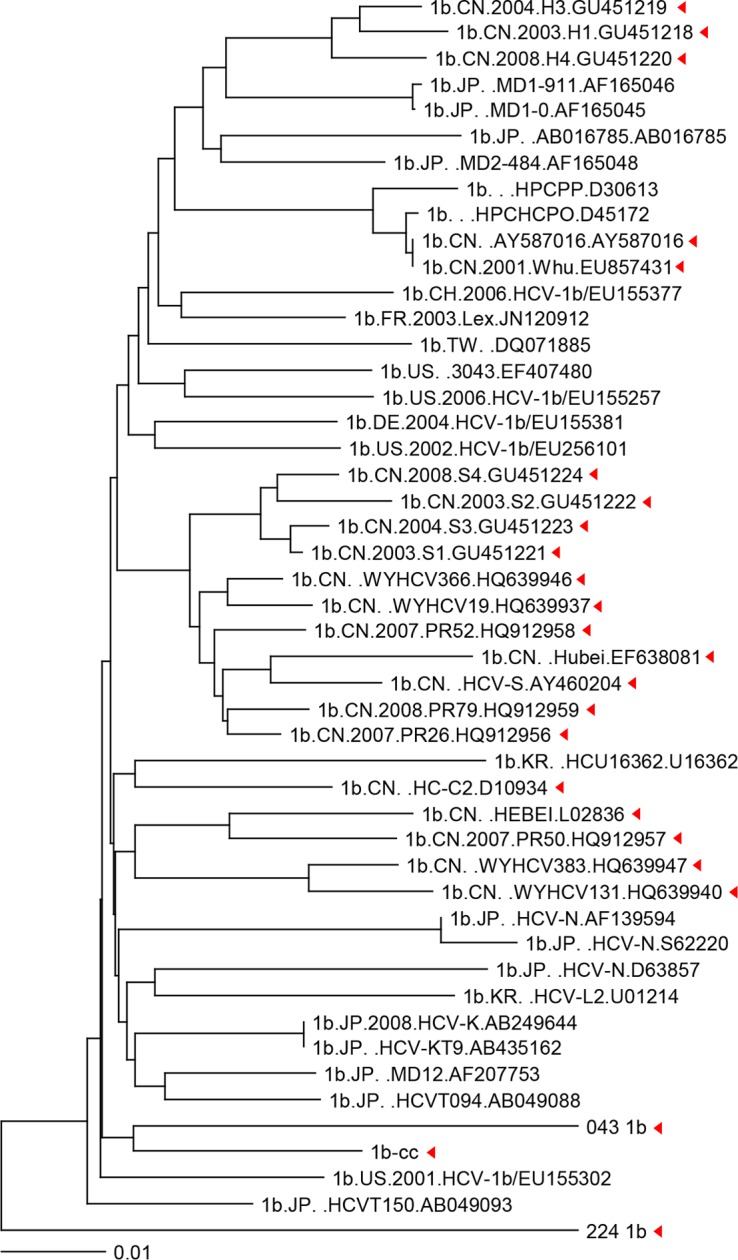
Chinese HCV 1b sequences are dispersed among global 1b sequences. Phylogenetic tree of 24 available Chinese HCV-1b strains and 24 examples from other geographic locations obtained from the HCV sequence database (hcv.lanl.gov/) and NCBI. Each red triangle represents one Chinese HCV-1b strain.

HCV-1b is the most prevalent HCV genotype in China and the majority of other areas in Asia. To generate a library of peptides to study HCV specific T cell response, all 392 global, including 24 Chinese, complete HCV-1b genomic sequences were downloaded from database and compared. Phylogenetic trees showed that Chinese HCV-1b sequences formed four clusters and dispersed among global HCV-1b sequences (Fig 1). A more complete sequence map was shown in S1 Fig. In order to map T cell responses to most HCV-1b variants in the patient population, we constructed a consensus HCV-1b sequence based on all 392 sequences, and synthesized an overlapping peptide library of 386 peptides that cover the entire length of HCV polyprotein.

Acute HCV infection progresses to either spontaneous recovery or chronic infection, the latter is frequently accompanied by an impaired or exhausted antigen-specific T cell response. To study the potential role of T cells in protective immune responses, we recruited 62 CHC-1b, 25 CHC-2a and 26 SPR DAA-naive patients into the study. Most of them were elderly with a median age of 50s, and approximately equal distribution of male and female (Table 1). Among them, a small proportion of HCV-1b individuals had previously been treated with the traditional interferon and ribavirin combination therapy, and some had undetected viremia levels (less than 15 IU/ml). None of them had received DAA based therapy.

**Table 1:** Characteristics of HCV infected patients used in T-cell epitope mapping.

Category	^a^CHC-1b (n = 61)	^b^CHC-2a (n = 24)	^c^SPR (n = 26)
Sex, female/male	32/29	13/11	16/10
Age, median (range)	51 (21–77)	58 (41–75)	56 (40–73)
^d^ALT (normal 7~40 U/L)			
<40 U/L	31	13	24
≥40 U/L	30	11	2
^e^AST (normal 13~35 U/L)			
<35 U/L	32	8	21
≥35 U/L	29	16	5
^f^Prior treatment, n (%)	30 (49.2)	0	0
Baseline viral load (IU/ml)			
Negative, n (%)	8 (13.1)	0	26 (100)
Positive, Mean (range)	6.35E+06 (292~3.77E+07)	1.88E+06 (7.07E+03~8.22E+06)	0

^a^ Chronic HCV-1b infection.

^b^ Chronic HCV-2a infection.

^c^ Spontaneous recovery.

^d^ Alanine aminotransferase.

^e^ Aspartate aminotransferase.

^f^ Interferon/ribavirin treatment.

**Fig 2:**
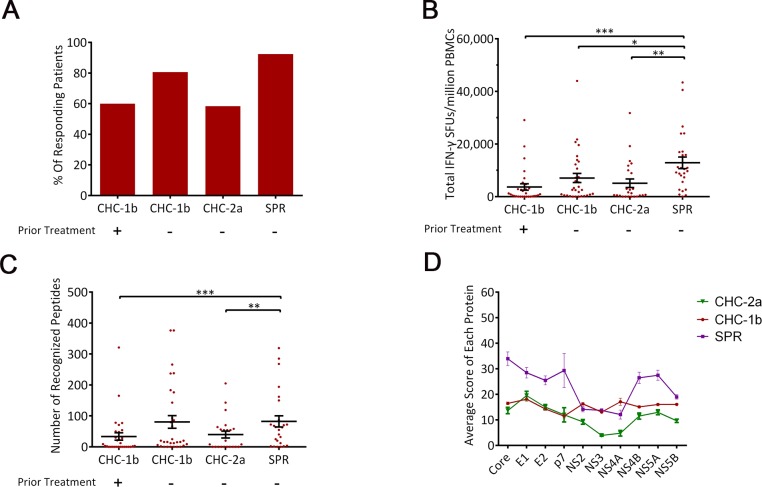
Stronger T cell responses correlate with better clinical outcome. Fresh PBMCs were isolated from each patient group (CHC-1b: chronic HCV-1b infection; CHC-2a: chronic HCV-2a infection; SPR: spontaneously recovery from acute HCV infection), and then used in the IFN-γ ELISPOT assay using HCV-1b peptide pools as stimulation. (A) The percentage of patients who had a positive response in different groups. (B) The magnitude of T cell responses in each patient group expressed as SFUs per million PBMCs. Each dot represents one donor. Mean ± SD were indicated by solid lines. (C) The total number of peptides recognized in each patient group. Mean ± SD were indicated by solid lines. (D) The average peptide-recognition score of each protein, as defined in the Materials and Methods. Mean ± SEM were derived from peptide scores of each protein. * represents p<0.05, ** represents p<0.01, and *** represents p<0.001.

**Fig 3:**
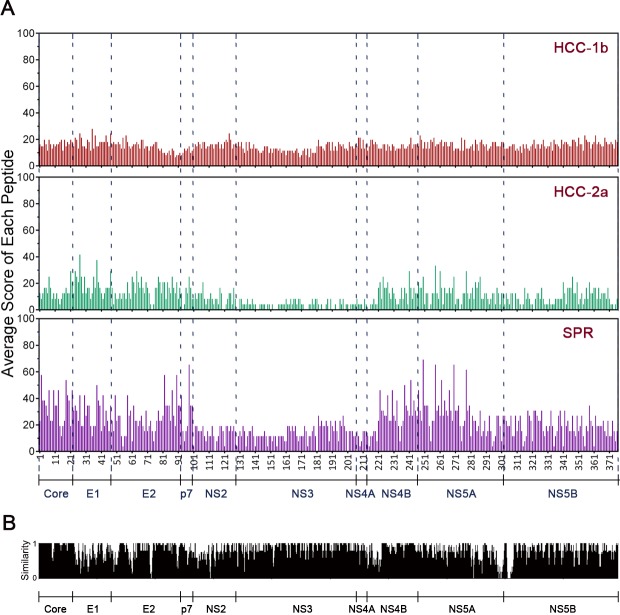
Spontaneous clearance showed stronger T cell responses than chronic infection. (A) A schematic illustration of average score of each peptide and each protein. In the epitope mapping ELISPOT assay, all positive peptides could be determined based on the peptide matrix method as described [18]. Each selected positive peptide was given a score of 100, and each negative peptide response was given a score of 0 in every ELISPOT assay to standardize the analyses. Then the average score of each peptide could be calculated with the total scores divided by the number of samples in each group. All peptides were shown along the abscissa according to their positions in the HCV polyprotein from N to C terminal. Red, CHC-1b. Green, CHC-2a. Purple, SPR. (B) The similarity score of each region among different HCV proteins. HCV-1b consensus polyprotein sequence was generated from 392 and HCV-2a from 33 available complete polyprotein sequences in the HCV database and NCBI using Megalign. The similarity score varied from 0 to 1, corresponding to amino acid identity. E, envelope protein. NS, non-structural protein.

PBMCs were freshly isolated from all patients and stimulated with 39 peptide pools comprised of 376 overlapping peptides covering the entire HCV polyprotein. HCV-specific T cell epitopes were mapped using the IFN-γ ELISPOT assay. Results showed that most peptide pools stimulated more IFN-γ production in SPR than both CHC-1b and CHC-2a patients (S2 Fig). Overall, SPR individuals also had significant higher response rate, greater magnitude, and broader antigen coverage than CHC-1b and CHC-2a patients (Fig 2A–2C). All HCV proteins contained T cell epitopes, and the highest score to each protein existed in the SPR group (Fig 2D). NS2, NS3, NS4A regions manifested relative lower antigenicity in SPR and CHC-2a groups. Scores of all proteins were low in the CHC-1b group, with no difference among HCV proteins. In addition, SPR recognized novel epitopes in Core, E1, E2, NS4B, NS5A regions that were not present in CHCs (Fig 3A). Because of the greater homology in Core, NS3, NS5A and NS5B in comparison to E1, E2, NS1, NS4A, the observed weak responses to NS3 and NS5B in HCV-2a patients were unlikely due to the use of HCV-1b peptides to map T cell epitopes (Fig 3B). In addition, neither age nor gender was associated with the magnitude of HCV-specific T cell responses (data not shown). Taking together, these results indicate that antigen-specific T cell responses may be one of the correlates of protection against the establishment of chronic HCV infection.

Since a vigorous T cell response has been associated with spontaneous resolution of acute HCV infection, we examined the specific sequences of immunodominant epitopes identified from SPRs. To avoid false negative results, we reanalyzed the data using mean+2SD (about 50 SFUs/million) of negative controls for positivity and identified a total of 39 immunodominant epitopes unique to SPRs. About half of them are derived from structural proteins Core, E1 and E2, another half from non-structural proteins p7, NS4B and NS5A. Some of them have high identity with HCV-2a peptides. Although some of the epitopes (16/39) have been reported previously, 23 of these epitopes (59%) have never been reported, most of these epitopes are within non-structural proteins (S1 Table).

### Lack of early T cell functional reconstitution during DAA therapy

**Fig 4:**
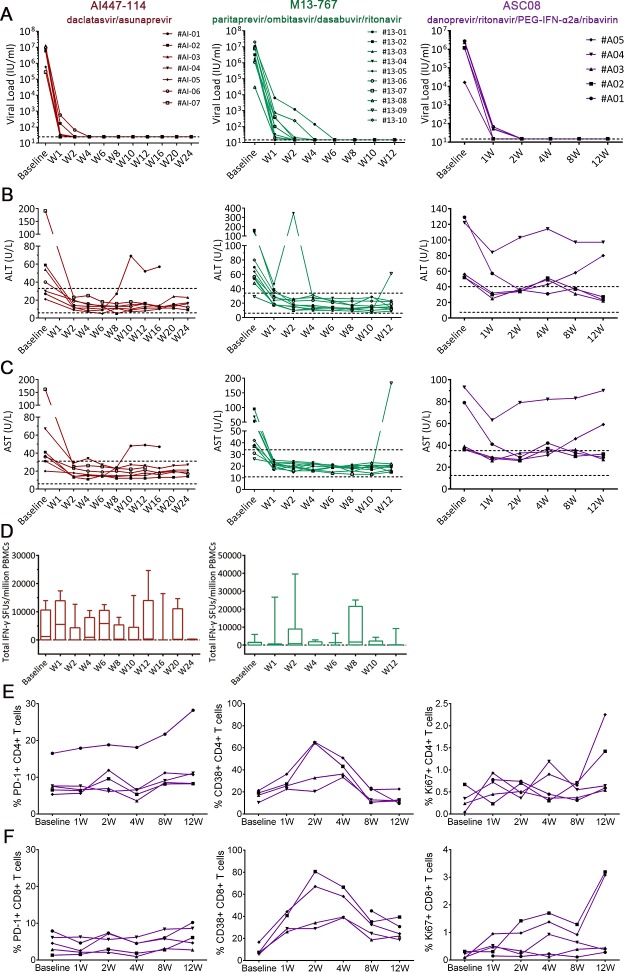
Sequential analysis of T cell activation during DAA therapy. The kinetics of viral load (A), ALT (B) and AST (C) in the blood were followed in 7, 10 and 5 patients enrolled in three clinical trials. “AI447-114”, a two-drug (daclatasvir/asunaprevir) DAA therapy; “M13-767”, a four-drug (paritaprevir/ombitasvir/dasabuvir/ritonavir) DAA therapy; “ASC08”, a four-drug (danoprevir/ritonavir/PEG-IFN-α2a/ribavirin) DAA therapy. (D) Sequential PBMC samples were obtained from the same patients of clinical trials “AI447-114” and “M13-767”. T cell response was measured with the IFN-γ ELISPOT assay. The total response at each time point was presented as SFUs/10^6^ PBMCs. The PD-1, CD38 and Ki67 expression levels on CD4+ (E) and CD8+ (F) T cells were determined using PBMCs from patients enrolled in trial “ASC08” with flow cytometry analysis. Baseline: first day before the beginning of treatment; Wn: n weeks of treatment.

DAA therapy offers an attractive option for the treatment of chronic HCV infection. To address the effects of DAA therapy on HCV-specific T cell response, we performed IFN-γ ELISPOT assay and flow cytometry analysis with freshly isolated PBMCs at different time points during treatment in 22 chronically HCV-1b infected patients underwent three different DAA regimens (Table 2). Viral load of all patients declined precipitously after the first week of treatment and then gradually decreased to below detection limits within 6 weeks (Fig 4A). Concurrently, there was a similar reduction of alanine aminotransferase (ALT) and aspartate aminotransferase (AST) levels in nearly all patients (Fig 4B and 4C). However, the magnitude of total IFN-γ response in all patient groups fluctuated between time points, with no discernable trend (Fig 4D). Therefore, although various DAA regimens efficiently cleared viruses from the blood and restored liver function test results, they did not improve antigen-specific T cell IFN-γ responses.

**Table 2:** Characteristics of HCV chronic-infected patients enrolled in clinical trials.

Category	DAA regiments		
	AI447-114 (daclatasvir/asunaprevir)	M13-767 (paritaprevir/ombitasvir/dasabuvir/ritonavir)	ASC08 (danoprevir/ritonavir/PEG-IFN-α2a/ribavirin)
Subject number	7	10	5
Sex, female/male	6/1	2/8	2/3
Age, median, n (range)	48 (27–50)	38 (22–60)	39 (25–56)
^a^Baseline stiffness			
<F2, n (%)	5 (71.4)	7 (70)	4 (80)
F2-F3, n (%)	2 (28.6)	3 (30)	1 (20)
^b^Prior treatment, n (%)	0	3 (30)	0

^a^ Based on clinical FibroScan value. <F2, 0~7.3kPa. F2-F3, 7.3~12.4kPa.

^b^ Interferon/ribavirin treatment.

Next, PBMCs isolated from patients enrolled in the clinical trial ASC08 were stained for PD-1, CD38 and Ki67 expression using specific antibodies, and then analyzed using flow cytometry. During DAA therapy, PD-1 expression levels on both CD4+ and CD8+ T cells did not change significantly. Instead, CD38 expression on both CD4+ and CD8+ T cells and Ki67 staining on CD8+ T cells showed a transient increase between 2 and 4 weeks of therapy (Fig 4E and 4F).

### Limited effect of anti-PD1 antibody on HCV-specific T cells

**Fig 5:**
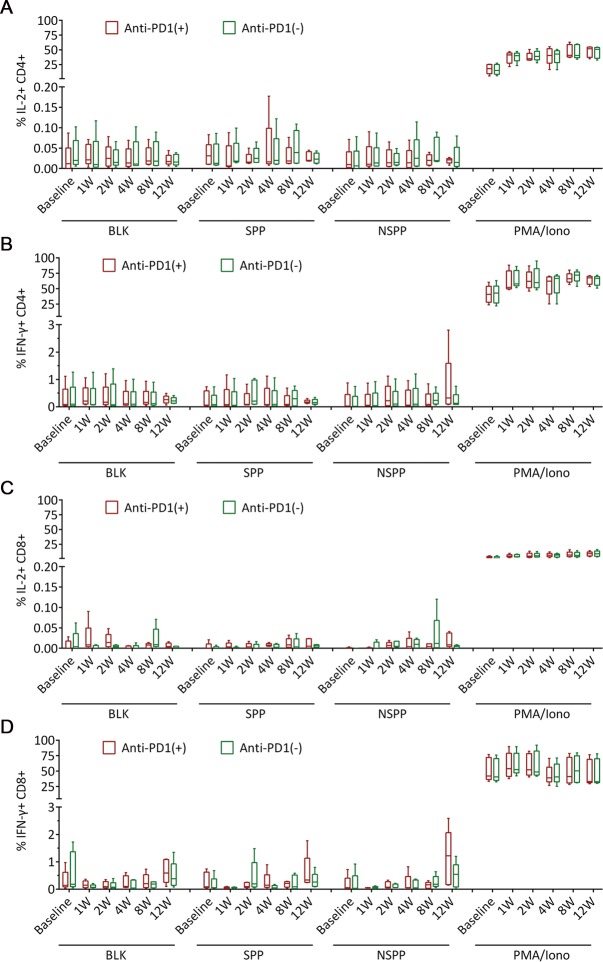
Limited effect of anti-PD1 antibody on the production of IL-2 and IFN-γ by HCV specific CD4+ and CD8+ T cells. PBMCs were obtained from 5 patients treated with danoprevir/ritonavir/PEG-IFN-α2a/RBV, and then stimulated with SPP, NSPP in the presence or absence of an anti-PD1 antibody. PMA and ionomycin (PMA/Iono) stimulated cells were used as positive controls, and unstimulated cells (BLK) were used as negative controls. Results showed (A) the percentage of IL-2+ CD4+ T cells, (B) the percentage of IFN-γ+ CD4+ T cells, (C) the percentage of IL-2+ CD8+ T cells, and (D) the percentage of IFN-γ+ CD8+ T cells at each time point. Red boxes indicated experiments with anti-PD-1 antibody during stimulation; green boxes indicated experiments without anti-PD-1 antibody during stimulation.

Since high PD-1 levels have been associated with the exhaustion of T cells during viral infections, we next examined whether blocking PD-1 *ex vivo* could rescue HCV-specific T cell functions. From the HCV-1b T cell epitope mapping experiments (Fig 2 and S1 Table), we have identified 39 immunodominant epitopes in SPR patients, and arranged them into two peptide pools: SPP containing 21 epitopes from structural proteins and NSPP containing 18 epitopes from non-structural proteins. PBMCs isolated from 5 patients in the ASC08 trial were stimulated with SPP and NSPP, respectively, with or without the addition of an anti-PD1 antibody. The production of IL-2 and IFN-γ from CD4+ and CD8+ T cell subsets after peptide stimulation was measured with the ICS assay. Results showed no significant production of either IL-2 or IFN-γ by both CD4+ (Fig 5A and 5B) and CD8+ T cells (Fig 5C and 5D) after stimulation with SPP or NSPP pools; additionally, blockade of PD-1 did not change significantly the production of IL-2 or IFN-γ by either subset of T cells (Fig 5A–5D). Thus, despite a transient activation of T cells (Fig 4E and 4F, middle panel), there was no early functional immune reconstitution of HCV-specific T cells during DAA therapy.

## Discussion

In this study, we comprehensively mapped T cell epitopes in HCV-infected patients in DAA treatment-naïve patients and found that a more favorable clinical outcome was associated with a better HCV-1b specific T cell response to multiple HCV proteins. Specifically, individuals with spontaneous clearance of viruses after acute infection had significantly higher response rate, greater magnitude of response, and broader antigen coverage than those who had become chronically infected. These data are in agreement with previous studies of patients infected by HCV- 1a and -3a, in whom T cell responses were associated with a spontaneous resolution of acute HCV infection [6, 13]. In chronic HCV-infected patients underwent DAA therapy, however, we found that although viruses were efficiently cleared from blood within the first few weeks of therapy, T cells were only transiently activated and there was no functional restitution of HCV-specific T cell responses.

The association between polyfunctional T cells and spontaneous resolution of HCV infection has been reported in some precious studies. However, none has systematically compared HCV-1b specific T cell responses between a large number of patients who either experienced spontaneous viral clearance after acute infection or became chronically infected. Using a unique synthetic HCV-1b peptide library covering the whole length of HCV polyprotein, we discovered that SPR patients have significant better antigen specific T cell response than both CHC-1b and CHC-2a infected patients.

Ideally, one would examine antigen specific T cell response using autologous viral sequences, but cost and lacking knowledge of viral sequences in those who have spontaneously recovered prohibit such an experimental approach. Although our peptide library was synthesized based on a HCV-1b consensus sequence, it enabled the detection of robust T cell responses in HCV-2a infected patients. In comparison, some HCV-1b chronically infected individuals had relatively low level of T cell responses despite being stimulated with HCV-1b peptides. These results suggest that the peptide library we used is an adequate tool for investigating the global HCV specific T cell responses. Furthermore, by comparing the consensus sequence of HCV-2a with the HCV-1b sequence used in this study (Fig 3B), we found that the weaker T cell response to NS proteins in HCV-2a (Figs 2D and 3A) were not associated with a lack of sequence homology within this region between the two viral subtypes. This suggests other mechanisms might be responsible for the observed differential T cell immune response between HCV-1b and HCV-2a infected individuals.

T cell epitopes in association with protective immune responses may inform the design of epitope-base subunit vaccines. In the past two decades, nearly 200 HCV specific T cell epitopes have been identified and deposited in the immune epitope database (IEDB) [23], some of them were linked to viral clearance. However, no one has systematically mapped T cell epitopes across the entire polyprotein of HCV-1b. By comprehensive T cell epitope mapping using peptide library, we have identified 39 immunodominant T cell epitopes in association with spontaneous recovery from HCV infection, many of these epitopes are within the Core, E1, E2, NS4B and NS5A regions (S1 Table). Despite some of the epitopes on structural proteins were reported previously, we have identified additional novel epitopes in the NS4B and NS5A regions. The high positivity of HCV-specific T cell responses observed in some individuals were based on analysis of results from a single assay, thus it should be interpreted judiciously. Further studies with more than one assay to confirm our findings are warranted. It is interesting to note that non-structural protein specific T cells have been linked to protection against HCV disease progression [24]. We also found that although all HCV proteins contain T cell epitopes, NS2, NS3 and NS4A regions manifested relatively lower antigenicity. This is different from some previous studies, in which most identified T cell non-structural protein epitopes were derived from NS3 and NS3, and these epitopes elicited more vigorous T cell responses than epitopes from other proteins [25, 26]. However, it has also been note previously that not all epitopes from NS3 are more immunodominant than other proteins in all patients [13] [27]. As is the case in our study, NS4B and NS5A specific T cell responses were stronger than those targeting NS3. This discrepancy may due to different peptide antigens used in the epitope mapping studies. Our peptide library was derived from a HCV-1b consensus sequence, which is not identical to any wild type viral sequence, and a single amino acid variation is known to significantly change the antigenicity of a peptide [27]. In addition, our study population is different from other patient cohorts, and thus the HCV quasispecies infecting these Chinese patients may be divergent from that infecting other patient population. Collectively, our HCV-1b epitope mapping results shared similarity but also revealed distinct differences with previous studies.

A number of T cell vaccines have already been tested in clinical trials with demonstrable induction of HCV specific CD4+ or CD8+ T cell responses [28], and these experimental HCV T cell vaccines often contain epitopes from the NS3 protein [29]. Our new data indicate that other novel epitopes might also be included to improve the current version of T cell vaccines.

Relatively a few studies have examined epitope-specific T cells in the context of DAA therapy. During the acute phase of viral infection, polyfunctional T cell responses were associated with viral clearance. Whereas in chronic infection, as antigen persists, antigen-specific T cells experienced various stages of dysfunction, including the loss of effector functions, an increased production of immunosuppressive cytokines, an elevated expression of inhibitory receptors, and finally exhaustion [30]. A previous report by Robert Thimme showed that DAA therapy restored *in vitro* proliferative capacity of HCV specific CD8+ T cells [12]. Another study reported memory T cell re-differentiation following DAA therapy [31]. In contrast, we found that even though all three DAA regimens led to a rapid decline of viral load, there was no augmentation of IFN-γ-production by HCV-specific T cells. This disagreement may be explained by several factors. First, the increased proliferation of T cells in previous studies could be resulted from the outgrowth of a small number of functional T cells rather than the reversion of T cell exhaustion [32]. Additionally, a recent *ex vivo* study using human samples have showed that DAA-induced HCV clearance does not completely restore the altered cytokine and chemokine milieu in chronic patients, including IFN-γ [33]. Of course, one limitation of our study is that we could not have followed the T cell responses in SVR patients for an extend period of time after the therapy, and therefore we can only conclude that there was a lack of early immune reconstitution in the patients after DAA-therapy. Besides, we used different DAA regiments on a different patient population and stimulated freshly isolated PBMCs with different HCV peptides for detecting T cell response *ex vivo*. Thus, HCV specific T cells could be completely exhausted during long-term chronic infection. Our results would suggest that early DAA treatment soon after infection might be needed to fully reconstitute T cell immune functions.

T cell functional changes during viral infection could be regulated by several mechanisms. Once T cells are committed to exhaustion during chronic infection, inhibitory receptors including PD-1 are induced and expressed at a relatively high level [30]. Initial partial exhaustion at the early stage of chronic infection gradually advances into severe exhaustion. In our study, all subjects enrolled in the DAA clinical trials had a long history of chronic HCV infection. Although T cells were transiently activated during the DAA therapy, the PD-1 expression on T cells remained unchanged at a relatively high level. Moreover, T cell functions were not restored by blocking the PD-1 pathway. These results suggest that long-term chronic infection may have led to a complete exhaustion of antigen specific T cells, and therefore early treatment is required for preserving T cell functions.

A limitation of our study is the modest sample size of 111 of DAA naïve HCV-infected patients and 22 patients on DAA. This is due partly to the difficulty in identifying SPR, and partly due to the lack of officially approved DAA in the Chinese clinics. By further increasing sample size, our observation on T cell response patterns might be confirmed. Another shortcoming of the current study is that IFN-α contained in one of the three DAA regiments may potentially affect T cell immune responses observed. A follow-up study including an IFN-α alone group should help to address this issue. However, whether it is ethically permissible to including such a control study group is uncertain, in the face of a wider accessibility to DAA.

In summary, we have performed the first, as far as we know, comprehensive comparison of T cell immune responses between Chinese patients with spontaneous recovery and chronic HCV-1b infection, in the presence or absence of DAA therapy. Our results suggest that a vigorous T cell response that targeting a breadth of HCV antigens might be a contributing factor to the resolution of acute HCV infection. Thus, peptide epitopes uniquely recognized in the SPR patients may be included in a vaccine against HCV. Additionally, neither the DAA treatment, nor blockade of PD-1 receptor fully reconstitutes T cell functions, implying exhaustion of HCV-specific T cells after a prolonged period of infection might have happened. Therefore, early DAA treatment would be need to fully preserve functional T cells.

## Supporting information

S1 Fig Dispersion of Chinese HCV-1b strains among all global HCV-1b complete genome sequences. Phylogenetic tree of 24 available Chinese HCV-1b strains among 392 global HCV-1b complete genome sequences available in the HCV sequence database (hcv.lanl.gov/). Each red triangle represents one Chinese HCV-1b strain.
(TIF)

S2 Fig The magnitude of IFN-γ responses to peptide pool. T cell responses were measured by IFN-γ ELISPOT assay using PBMC obtained from different patient groups. PHA stimulated cells were used as positive controls, and unstimulated cells (BLK) were used as negative controls. (A) Representative sample wells of IFN-γ specific spots in 1 HC, 1 CHR-1b, 1 CHR-2a and 1 SPR subject after stimulation with 4 peptide pools (P4, P12, P20, P28, P36) or PHA or unstimulated (BLK). The magnitude of response to each peptide pool in 3 HC (B), 61 CHR-1b (C), 24 CHR-2a (D) and 26 SPR (E) were shown. Each dot represented one subject. Mean ± SEM were indicated by a solid line and error bars for each peptide pool.
(TIF)

S1 Table Immunodominant HCV-1b T cell epitopes identified. (PDF)
