# Supplementary figures and images for: Comprehensive mapping of antigen specific T cell responses in hepatitis C virus infected patients with or without spontaneous viral clearance
Source: PLoS One. 2017 Feb 7;12(2):e0171217. doi: 10.1371/journal.pone.0171217 (PMC5295680; doi:10.1371/journal.pone.0171217)

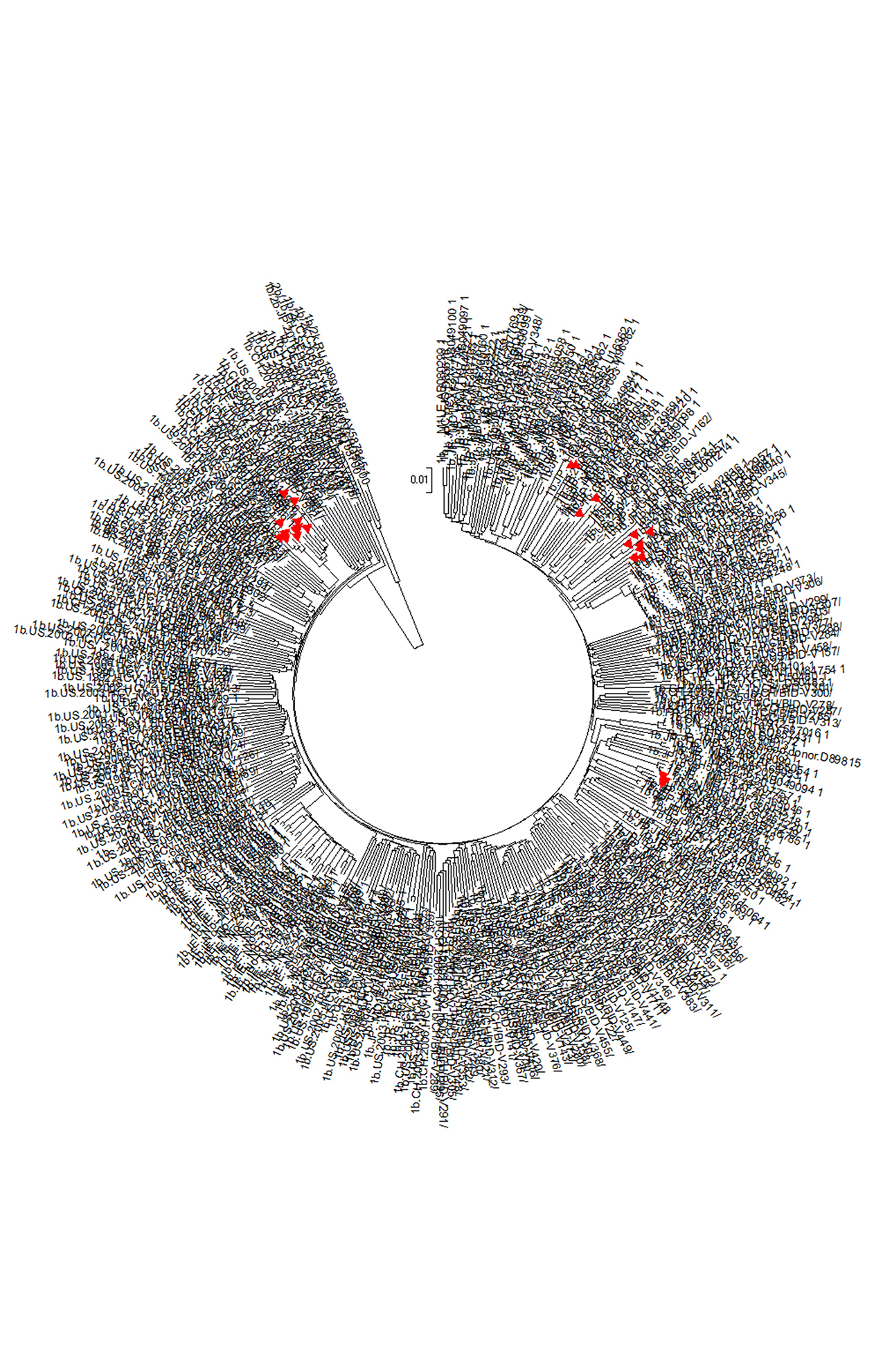

Supplement: S1 Fig — Phylogenetic tree of 24 available Chinese HCV-1b strains among 392 global HCV-1b complete genome sequences available in the HCV sequence database (hcv.lanl.gov/). Each red triangle represents one Chinese HCV-1b strain. (TIF) [file pone.0171217.s001.tif]

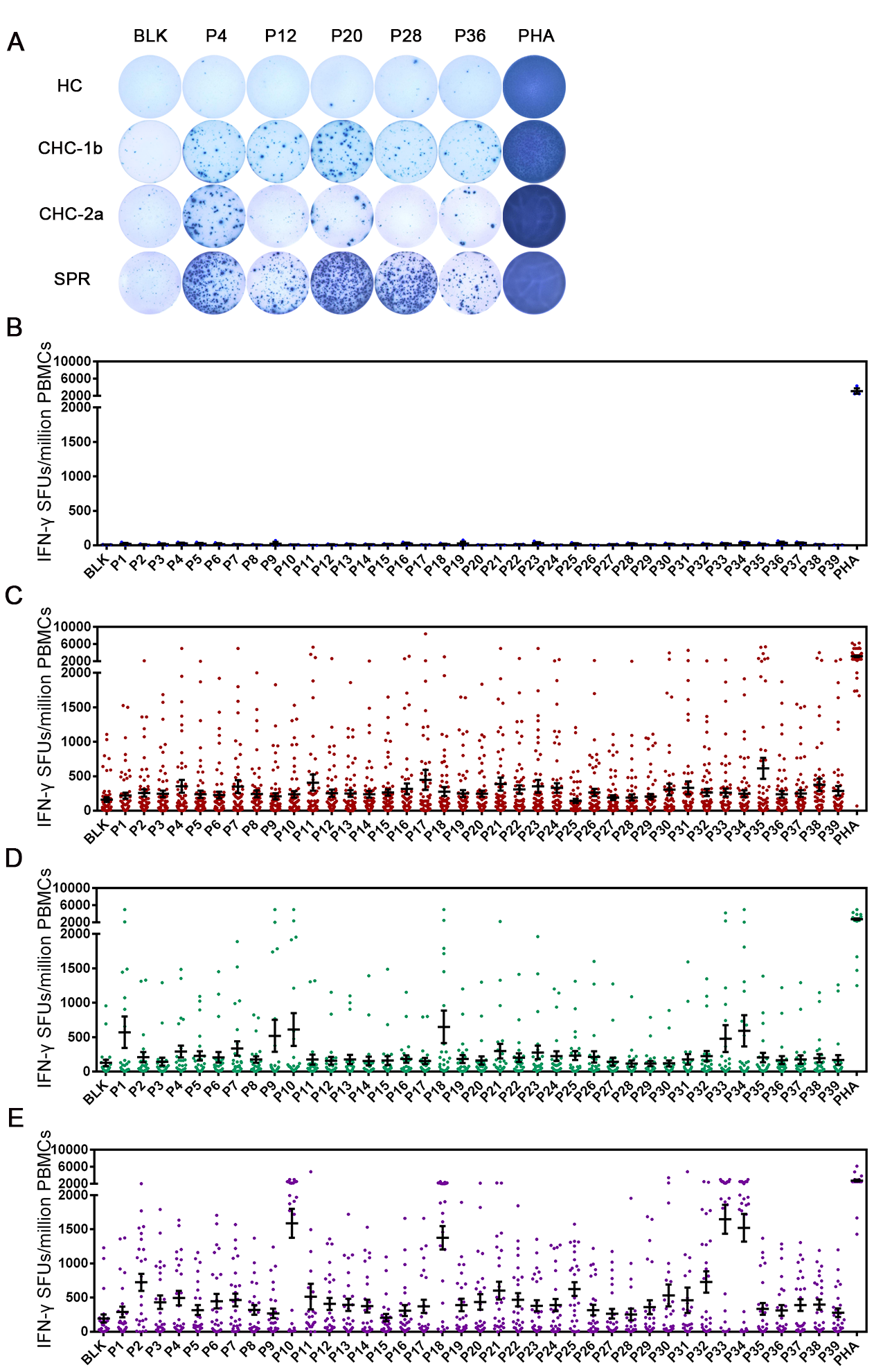

Supplement: S2 Fig — T cell responses were measured by IFN-γ ELISPOT assay using PBMC obtained from different patient groups. PHA stimulated cells were used as positive controls, and unstimulated cells (BLK) were used as negative controls. (A) Representative sample wells of IFN-γ specific spots in 1 HC, 1 CHR-1b, 1 CHR-2a and 1 SPR subject after stimulation with 4 peptide pools (P4, P12, P20, P28, P36) or PHA or unstimulated (BLK). The magnitude of response to each peptide pool in 3 HC (B), 61 CHR-1b (C), 24 CHR-2a (D) and 26 SPR (E) were shown. Each dot represented one subject. Mean ± SEM were indicated by a solid line and error bars for each peptide pool. (TIF) [file pone.0171217.s002.tif]
