# Supplementary material for: Comprehensive mapping of antigen specific T cell responses in hepatitis C virus infected patients with or without spontaneous viral clearance
Source: PLoS One. 2017 Feb 7;12(2):e0171217. doi: 10.1371/journal.pone.0171217 (PMC5295680; doi:10.1371/journal.pone.0171217)
Supplement: S1 Table — (PDF) [file pone.0171217.s003.pdf]

Supporting Information

S1 Table. Immunodominant HCV-1b T cell epitopes identified.

	Core (1-18) *Score 69.23	Core (9-26) Score 69.23	Core (17-34) Score 57.69	Core (49-66) Score 53.85	Core (65-82) Score 65.38	Core (73-90) Score 65.38
HCV-1b Peptide	MSTNPKPQRKTKRNTNRR	RKTKRNTNRRPQDVKFPG	RRPQDVKFPGGGQIVGGV	TRKTSERSQPRGRRQPIP	IPKARRPEGRTWAQPGYP	GRTWAQPGYPWPLYGNEG
HCV-2a Peptide	MSTNPKPQRKTKRNTNRR	RKTKRNTNRRPQDVKFPG	RRPQDVKFPGGGQIVGGV	TRKTSERSQPRGRRQPIP	IPKDRRSTGKSWGKPGYP	GKSWGKPGYPWPLYGNEG
Known Epitope	MSTNPKPQRKTKRNTNRRPQ[1]	TKRNTLRRPQDVRF[2]	QDVRFPGGGQIVGG[2]		KARQPEGRAWAQPG[2]	GRAWAQPGYPWPLYGNEGL[3]

	Core (97-114) Score 53.85	Core (137-154) Score 73.08	Core (145-162) Score 53.85	Core (153-170) Score 53.85	Core (161-178) Score 61.54	Core (169-186) Score 57.69
HCV-1b Peptide	LLSPRGSRPSWGPSDPRR	IPLVGAPLGGAARALAHG	GGAARALAHGVRVLEDGV	HGVRVLEDGVNYATGNLP	GVNYATGNLPGCSFSIFL	LPGCSFSIFLLALLSCLT
HCV-2a Peptide	LLSPRGSRPSWGPTDPRH	IPVVGAPLGGVARALAHG	GGVARALAHGVRVLEDGV	HGVRVLEDGVNYATGNLP	GVNYATGNLPGCSFSIFL	LPGCSFSIFLLALLSCIT
Known Epitope	RGSRPSWGPTDPRRR[4]	LVGAPLGGAARAL[4]	ALAHGVRVL[4]			SFSIFLLALL[5]<br>IFLLALLSCL[5]

	E1 (225-242) Score 57.69	E1 (233-250) Score 57.69	E1 (297-314) Score 65.38	E2 (457-474) Score 53.85	E2 (641-658) Score 73.08	E2 (649-666) Score 69.23
HCV-1b Peptide	GCVPCVRENNSSRCWVAL	NNSSRCWVALTPTLAARN	RHETVQDCNCSIYPGHVS	ASCRPIDKFAQGWGPITY	NAACNWTRGERCDLEDRD	GERCDLEDRDRSELSPLL
HCV-2a Peptide	GCVPCEKVGNTSRCWIPV	XNTSRCWIPVSPNVAVRQ	HHWFVQECNCSIYPGTIT	SACRNIEAFRIGWGTLQY	TAACNFTRGDRCDLEDRD	GDRCDLEDRDRSQLSPLL
Known Epitope	VPCVREGNVSRCWVA[6]	RCWVAMTPTVATRDG[6]	RHWTTQGCNCSIYPG[6]		WTRGERCNL[7]	

	E2 (689-706) Score 57.69	E2 (705-722) Score 73.08	E2 (713-730) Score 69.23
HCV-1b Peptide	LIHLHQNIVDVQYLYGVG	VGSAVVSFAIKWEYVLLL	AIKWEYVLLLFLLLADAR
HCV-2a Peptide	LLHLHQNIVDVQYMYGLS	LSPALTKYVVRWEWVVLL	VVRWEWVVLLFLLLADAR
Known Epitope			WEYVLLLFL[7]

	p7 (777-794) Score 76.92	NS4B (1761-1778) Score 65.38	NS4B (1769-1786) Score 57.69	NS4B (1825-1842) Score 61.54	NS4B (1833-1850) Score 65.38	NS4B (1897-1914) Score 69.23

HCV-1b Peptide	YIKGRLVPGAAYALYGVW	WAKHMWNFISGIQYLAGL	ISGIQYLAGLSTLPGNPA	AASAFVGAGIAGAAVGSI	GIAGAAVGSIGLGKVLVD	VCAAILRRHVGPGEGAVQ
HCV-2a Peptide	YIKGRAVPLATYSLTGLW	WAKHMWNFISGIQYLAGL	ISGIQYLAGLSTLPGNPA	GATGFVVSGLVGAAVGSI	GLVGAAVGSIGLGKVLVD	ICAAILRRHVGPGEGAVQ
Known Epitope						

	NS4B (1921-1938) Score 80.77	NS4B (1929-1946) Score 61.54	NS5A (1969-1986) Score 53.85	NS5A (1985-2002) Score 76.92	NS5A (1993-2010) Score 80.77	NS5A (2057-2074) Score 76.92
HCV-1b Peptide	AFASRGNHVSPTHYVPES	VSPTHYVPESDAAARVTQ	STPCSGSWLRDVWDWICT	CTVLTDFKTWLQSKLLPR	TWLQSKLLPRLPGVPFLS	HGTFPINAYTTGPCTPSP
HCV-2a Peptide	AFASRGNHVAPTHYVTES	VAPTHYVTESDASQRVTQ	PIPCSGSWLRDVWDWVCT	CTILTDFKNWLTSKLFPK	NWLTSKLFPKMPGLPFIS	QGTFPINCYTEGQCVPKP
Known Epitope				VLSDFKTWL [8]<br>VLTDFKTWL [9],[10]		

	NS5A (2081-2098) Score 73.08	NS5A (2089-2106) Score 57.69	NS5A (2129-2146) Score 53.85	NS5A (2145-2162 Score 69.23	NS5A (2153-2170) Score 73.08	NS5A (2217-2234) Score 73.08
HCV-1b Peptide	ALWRVAAEEYVEVTRVGD	EYVEVTRVGDFHYVTGMT	RLHRYAPACKPLLRDEVA	VAFQVGLNQYLVGSQLPC	QYLVGSQLPCEPEPDVAV	THHDSPDADLIEANLLWR
HCV-2a Peptide	AIWRVAASEYAEVTQHGS	EYAEVTQHGSYSYITGLT	QIHRFAPTPKPFFRDEVS	VSFCVGLNSFVVGSQLPC	SFVVGSQLPCDPEPDTDV	THGKTYDVDMVDANLFMG
Known Epitope			RYAPACKPL [5],[11],[12]			

[a] Score, average score of each peptide above 50 in peptide mapping. Red and blue colors represent nonidentical sites.

Supplementary References

1. MacDonald AJ, Duffy M, Brady MT, McKiernan S, Hall W, Hegarty J, et al. CD4 T helper type 1 and regulatory T cells induced against the same epitopes on the core protein in hepatitis C virus-infected persons. The Journal of infectious diseases. 2002;185(6):720-7.

2. Lasarte JJ, Garcia-Granero M, Lopez A, Casares N, Garcia N, Civeira MP, et al. Cellular immunity to hepatitis C virus core protein and the response to interferon in patients with chronic hepatitis C. Hepatology. 1998;28(3):815-22.

3. Humphreys IS, von Delft A, Brown A, Hibbert L, Collier JD, Foster GR, et al. HCV genotype-3a T cell immunity: specificity, function and impact of therapy. Gut. 2012;61(11):1589-99.

4. Lohr HF, Schlaak JF, Kollmannsperger S, Dienes HP, Meyer zum Buschenfelde KH, Gerken G. Liver-infiltrating and circulating CD4+ T cells in chronic hepatitis C: immunodominant epitopes, HLA-restriction and functional significance. Liver. 1996;16(3):174-82.

5. Takao Y, Yamada A, Yutani S, Takedatsu H, Ono T, Etoh K, et al. Identification of new immunogenic peptides in conserved regions of hepatitis C virus (HCV) 1b with the potentiality to generate cytotoxic T lymphocytes in HCV1b(+) HLA-A24(+) patients. Hepatology research : the official journal of the Japan Society of Hepatology. 2007;37(3):186-95.

6. Sarobe P, Jauregui JI, Lasarte JJ, Garcia N, Civeira MP, Borras-Cuesta F, et al. Production of interleukin-2 in response to synthetic peptides from

hepatitis C virus E1 protein in patients with chronic hepatitis C: relationship with the response to interferon treatment. Journal of hepatology. 1996;25(1):1-9.

7. Guo Z, Zhang H, Rao H, Jiang D, Cong X, Feng B, et al. DCs pulsed with novel HLA-A2-restricted CTL epitopes against hepatitis C virus induced a broadly reactive anti-HCV-specific T lymphocyte response. PloS one. 2012;7(6):e38390.

8. Urbani S, Uggeri J, Matsuura Y, Miyamura T, Penna A, Boni C, et al. Identification of immunodominant hepatitis C virus (HCV)-specific cytotoxic T-cell epitopes by stimulation with endogenously synthesized HCV antigens. Hepatology. 2001;33(6):1533-43.

9. Penna A, Pilli M, Zerbini A, Orlandini A, Mezzadri S, Sacchelli L, et al. Dysfunction and functional restoration of HCV-specific CD8 responses in chronic hepatitis C virus infection. Hepatology. 2007;45(3):588-601.

10. Masalova OV, Lesnova EI, Pichugin AV, Melnikova TM, Grabovetsky VV, Petrakova NV, et al. The successful immune response against hepatitis C nonstructural protein 5A (NS5A) requires heterologous DNA/protein immunization. Vaccine. 2010;28(8):1987-96.

11. Mashiba T, Udaka K, Hirachi Y, Hiasa Y, Miyakawa T, Satta Y, et al. Identification of CTL epitopes in hepatitis C virus by a genome-wide computational scanning and a rational design of peptide vaccine. Immunogenetics. 2007;59(3):197-209.

12. Yutani S, Yamada A, Yoshida K, Takao Y, Tamura M, Komatsu N, et al. Phase I clinical study of a personalized peptide vaccination for patients

infected with hepatitis C virus (HCV) 1b who failed to respond to interferon-based therapy. Vaccine. 2007;25(42):7429-35.
